# The mitochondrial logic of inflammaging: how energy imbalance drives fibroblast SASP and tissue-specific aging

**DOI:** 10.3389/fimmu.2026.1881243

**Published:** 2026-07-01

**Authors:** Yu Huan, Qiqi Wang, Rongkaixuan Fang, Yue Sun, Meiya Suo, Chenghong Cao, Shuya Zhang, Lan A, Wenzhou Xu

**Affiliations:** 1 Department of Periodontology, Hospital of Stomatology, Jilin University, Changchun, Jilin, China; 2 Jilin Provincial Key Laboratory of Tooth Development and Bone Remodeling, Hospital of Stomatology, Jilin University, Changchun, Jilin, China; 3 Department of Oral Implantology, Hospital of Stomatology, Jilin University, Changchun, Jilin, China

**Keywords:** cellular senescence, fibroblasts, inflammaging, innate immune signaling, metabolic reprogramming, mitochondrial dysfunction, senescence-associated secretory phenotype (SASP)

## Abstract

The conversion of metabolic disequilibrium into chronic inflammatory signaling represents a central and actively investigated question in ageing biology. Among stromal cells, fibroblasts are key effectors of tissue remodeling and inflammation, acquiring a senescence-associated secretory phenotype (SASP) that sustains age-related pathology. Here, we delineate a mechanistic framework in which disruption of energy homeostasis drives mitochondrial dysfunction, innate immune activation, and SASP secretion. Mitochondria act as metabolic sentinels that sense energetic stress through altered AMP/ATP and NAD^+^/NADH ratios, leading to the generation of mitochondrial danger signals—reactive oxygen species (mtROS) and mitochondrial DNA (mtDNA). These signals converge on canonical immune pathways, including the cGAS–STING axis, NLRP3 inflammasome, and NF-κB signaling, thereby converting metabolic distress into persistent pro-inflammatory output. Using periodontal ligament fibroblasts as a disease-relevant model, we highlight how microbial biofilm exposure induces mitochondrial metabolic reprogramming that amplifies fibroblast SASP, promotes osteoclastogenesis, extracellular-matrix degradation, and alveolar bone resorption. At the transcriptional level, regulatory networks involving NF-κB, C/EBPβ, STATs, and the mTOR–AMPK hub integrate mitochondrial signals to sustain inflammatory senescence. We propose that restoring mitochondrial metabolic homeostasis serves as a highly promising strategy to break the self-perpetuating cycle in which energy imbalance triggers SASP activation, which in turn contributes to chronic inflammation. Researchers must first characterize the tissue-specific mitochondrial signatures of SASP. Subsequently, developing precise, lesion-targeted metabolic interventions will open new avenues for mitigating inflammaging and rejuvenating stromal function across ageing tissues.

## 1 Introduction

With the continuous rise of the global ageing population, the incidence of age-associated disorders such as atherosclerosis and type 2 diabetes is steadily increasing. These conditions share a common pathogenic denominator—persistent, low-grade systemic inflammation, often referred to as inflammageing (1–3). Unlike classical inflammation triggered by pathogens, inflammageing arises from the progressive accumulation and pathological amplification of endogenous danger signals generated during organismal ageing.

Cellular senescence represents a central hallmark of ageing. When cells irreversibly exit the cell cycle, they adopt a senescence-associated secretory phenotype (SASP), characterized by the synthesis and release of extracellular matrix–degrading enzymes, chemokines, and proinflammatory cytokines. Through paracrine signaling, these factors propagate tissue remodeling, chronic inflammation, and disease progression (4–7). Fibroblasts, as principal stromal components across multiple tissues, play indispensable roles in this process. Unlike dermal or cardiac fibroblasts, which primarily undergo fibrotic scarring and excessive collagen deposition during aging, periodontal ligament fibroblasts (PDLFs) inhabit a uniquely dynamic mechanical and microbial microenvironment. This predisposes PDLFs to a highly degradative SASP profile characterized by excessive osteoclastogenic factor secretion and matrix metalloproteinase hyperactivation. Thus, PDLFs serve as an optimal disease-relevant model to study how metabolic stress drives tissue-destructive inflammatory aging, providing insights fundamentally distinct from the fibrotic responses of lung or synovial fibroblasts (8–10).

Although the regulatory mechanisms of SASP have been extensively investigated, previous reviews predominantly focus on broad inflammatory signaling or generalized immune cell models. In contrast, this review introduces a novel perspective by positioning mitochondrial energy imbalance as the fundamental upstream driver of the fibroblast-specific SASP program. Furthermore, we uniquely emphasize tissue-specific aging by utilizing periodontal ligament fibroblasts as a translational model, linking localized metabolic dysfunction to extracellular matrix degradation and alveolar bone resorption. However, how mitochondrial dysfunction in ageing fibroblasts coordinates metabolic perturbation with sustained SASP expression remains to be systematically elucidated.

In this review, we introduce a conceptual framework that links metabolic imbalance, mitochondrial injury, and mitochondrial signaling activation to fibroblast SASP expression. By emphasizing the molecular logic of mitochondrial regulation in SASP induction, this work aims to fill a critical gap in current understanding and to provide mechanistic insights and therapeutic implications for tissue-specific inflammatory ageing and related pathologies.

## 2 Energetic imbalance and mitochondrial dysfunction in fibroblast senescence

### 2.1 Initiation of mitochondrial decline and cellular energy imbalance

During ageing, fibroblasts undergo extensive metabolic remodeling, prominently manifested as progressive mitochondrial dysfunction. As the cellular powerhouses, mitochondria generate ATP primarily through oxidative phosphorylation (OXPHOS). However, the accumulation of mitochondrial DNA mutations, elevated oxidative stress, and impaired mitophagy collectively reduce OXPHOS efficiency and ATP production (11, 12).

Importantly, mitochondria are not merely energy converters but also vital metabolic sensors that monitor cellular energy status and flux. When ATP synthesis declines, mitochondria transform energy deficiency into multilayered signaling cues, biochemically reflected by an increased AMP/ATP ratio and a decreased NAD^+^/NADH ratio (13–15). These changes indicate a collapse of metabolic equilibrium and trigger adaptive stress signaling, laying the foundation for the metabolic-to-inflammatory transition characteristic of senescence.

### 2.2 AMPK and NAD^+^/sirtuin pathways as energy-sensing signaling axes

As a cellular energy sentinel, AMP-activated protein kinase (AMPK) is among the earliest responders to energy depletion. Rising AMP levels allosterically activate AMPK, which then orchestrates multiple energy-conserving and compensatory pathways. In fibroblasts, phosphorylated AMPK enhances fatty acid oxidation and glycolysis to replenish ATP rapidly (16–18). Moreover, AMPK induces the activation of peroxisome proliferator–activated receptor gamma coactivator-1α (PGC-1α), thereby promoting mitochondrial biogenesis to counteract functional decline (19, 20). In parallel, AMPK phosphorylates Raptor within the mTORC1 complex, inhibiting its kinase activity and suppressing energy-intensive anabolic processes such as protein synthesis (21–23).

The role of AMPK in inflammation is context-dependent, capable of promoting or suppressing inflammatory responses depending on the cellular milieu. Its anti-inflammatory action involves phosphorylation of the NF-κB p65 subunit and activation of deacetylases such as SIRT1, which repress proinflammatory gene transcription (24–27). However, under chronic energy disequilibrium associated with ageing, prolonged AMPK activation may induce adaptive feedback responses. The sustained suppression of anabolic metabolism, combined with energy stress, may prime cells toward a senescence-associated secretory phenotype (SASP) (28, 29).

NAD^+^ acts as an essential cofactor in glycolysis, the tricarboxylic acid (TCA) cycle, and β-oxidation, and its depletion is a hallmark of disrupted energy homeostasis. The sirtuin family of NAD^+^-dependent deacetylases governs mitochondrial function, stress responses, and longevity. Age-related upregulation of CD38 accelerates NAD^+^ degradation, leading to severe NAD^+^ depletion that directly inhibits SIRT1 and SIRT3 activities (30–34).

**Figure 1:**
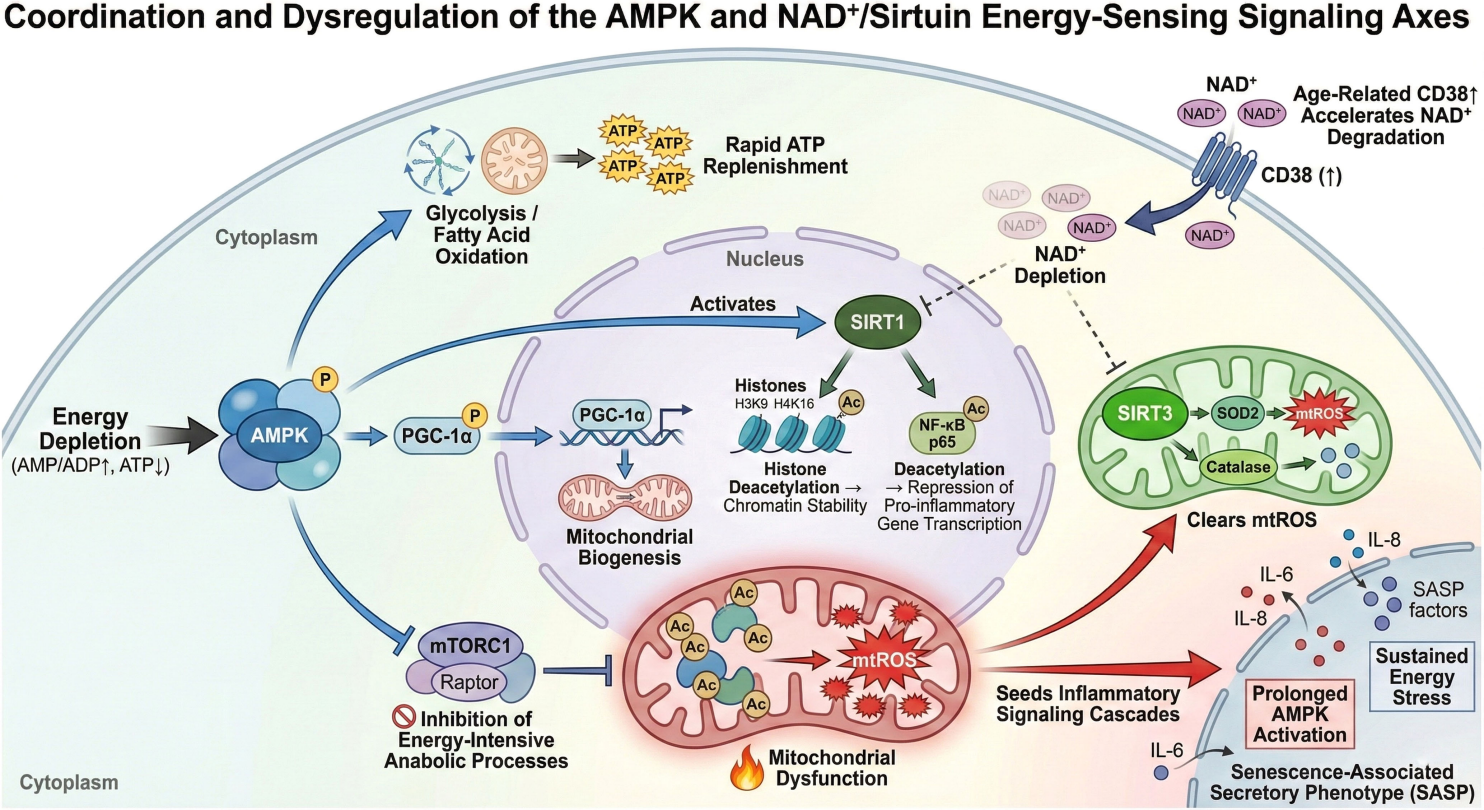
Coordination and Dysregulation of the AMPK and NAD+/Sirtuin Energy-Sensing Signaling Axes.

SIRT3, localized in mitochondria, deacetylates and activates antioxidant enzymes such as SOD2 and catalase, forming a major defense against mitochondrial ROS. Nuclear SIRT1 catalyzes histone deacetylation (e.g., H1K26, H3K9, H4K16) and regulates transcription factors such as p53, NF-κB p65, and FOXOs, thereby maintaining chromatin stability and modulating inflammatory responses. Inhibition of SIRT3 leads to hyperacetylation of mitochondrial proteins, including OXPHOS components, which exacerbates mitochondrial dysfunction and mtROS accumulation, seeding inflammatory signaling cascades (35, 36)(**Figure 1**).

### 2.3 Metabolic signaling and the bioenergetic basis of SASP expression

Under hypoxic or metabolically compromised conditions, cytosolic succinate levels rise markedly. Succinate competitively inhibits prolyl hydroxylases, preventing the degradation of HIF-1α, and thus activates proinflammatory gene expression such as IL-1β even under normoxic conditions (37, 38). Moreover, succinate promotes mtROS generation and directly stimulates NLRP3 inflammasome assembly.

α-Ketoglutarate (α-KG), a crucial TCA intermediate, acts as an essential retrograde messenger from mitochondria to the nucleus. As an obligate cofactor for ten-eleven translocation (TET) DNA demethylases and Jumonji C (JmjC)-domain-containing histone demethylases, α-KG directly links mitochondrial metabolic flux to nuclear epigenetic remodeling. During senescence, mitochondrial dysfunction depletes mitochondrial α-KG pools, shifting the α-KG/succinate ratio. This metabolic starvation inhibits demethylase activity, resulting in the hypermethylation of repressive chromatin marks at anti-inflammatory gene loci, while paradoxically permitting the epigenetic derepression of key SASP promoters. Consequently, α-KG acts as a direct molecular bridge translating mitochondrial metabolic stress into sustained inflammatory transcription (39, 40).

Through continuous monitoring of AMP/ATP and NAD^+^/NADH ratios, as well as TCA-cycle metabolite levels, mitochondria precisely integrate metabolic and stress cues. This integration forms an AMPK–Sirtuin–metabolite signaling axis that transforms fibroblast energetic imbalance into sustained proinflammatory activation. Consequently, metabolic insufficiency not only reflects diminished energy production but also initiates a persistent feed-forward signal that activates downstream inflammatory cascades and establishes the metabolic foundation for SASP expression.

### 2.4 Mitochondria-associated endoplasmic reticulum membranes as upstream signaling hubs

Prior to the cytosolic release of mitochondrial danger signals, inter-organelle communication at mitochondria-associated endoplasmic reticulum membranes (MAMs) serves as a critical upstream regulatory node. Aging-induced structural alterations in MAMs disrupt tethering complexes (eg, IP3R-GRP75-VDAC1), leading to pathological Ca2+ transfer from the ER to mitochondria (41). This mitochondrial Ca2+ overload simultaneously dissipates the mitochondrial membrane potential and accelerates the overproduction of mtROS (42). Furthermore, disrupted MAM integrity exacerbates ER stress and facilitates the propagation of phospholipid peroxidation (43). Crucially, MAMs act as necessary spatial platforms for the assembly of the NLRP3 inflammasome and the initiation of cGAS-STING signaling upon mtDNA leakage. Thus, MAM dysfunction functions as a critical intermediate, completing the sequence from energetic imbalance to the upstream generation of mitochondrial danger signals, which subsequently drive innate immune activation and the fibroblast SASP.

## 3 Mitochondria-derived signals: triggers of SASP activation

### 3.1 Mitochondrial ROS as central signaling mediators of multidimensional inflammatory activation

In fibroblasts under ageing or stress conditions, dysfunctional mitochondria are not passive victims of energetic failure but active hubs for inflammatory regulation. Damaged mitochondria release a set of mitochondrial-derived damage-associated molecular patterns (mtDAMPs), which are recognized by cytosolic pattern recognition receptors (PRRs) and thereby initiate and amplify innate immune signaling. This represents a key molecular switch that triggers the senescence-associated secretory phenotype (SASP) (44).

Among these mtDAMPs, mitochondrial reactive oxygen species (mtROS) function as both signal amplifiers and primary inducers of inflammation. When mitochondrial function declines, increased electron leakage from the respiratory chain leads to mtROS overproduction—predominantly superoxide anions. Senescent cells consistently exhibit higher mtROS levels than young cells due to chronic oxidative stress (45).

Excessive mtROS not only oxidatively damage lipids, proteins, and nucleic acids but also act as pivotal signaling intermediates. Specifically, mtROS-induced oxidative modification of the IκB kinase (IKK) complex activates its catalytic subunits, resulting in IκBα phosphorylation and subsequent ubiquitin-mediated degradation. This process liberates NF-κB dimers, allowing their nuclear translocation and transcriptional activation of proinflammatory genes such as *IL-6*, *IL-8*, and *TNF-α*. Additionally, mtROS directly oxidize IκBα, weakening its interaction with NF-κB and further facilitating NF-κB activation. Redox-sensitive kinases such as apoptosis signal–regulating kinase 1 (ASK1) and p38 MAPK are also stimulated by mtROS, thereby intensifying inflammatory signal transduction (46).

Crucially, mtROS serve as indispensable activators of the NLRP3 inflammasome, a multiprotein complex composed of the NLRP3 receptor, the adaptor ASC, and the effector pro–caspase-1. Elevated mtROS levels promote the interaction between NLRP3 and the kinase NEK7, enabling NLRP3 oligomerization and recruitment of ASC and pro–caspase-1, leading to inflammasome assembly and activation (47). Activated caspase-1 cleaves the precursors pro–IL-1β and pro–IL-18 into their mature, bioactive cytokine forms. These cytokines are released via pyroptosis or unconventional secretion pathways, strongly amplifying local and systemic inflammation. In senescent fibroblasts, persistent NLRP3 inflammasome activation constitutes a major mechanism driving the sustained release of IL-1β and other proinflammatory mediators (48).

### 3.2 Mitochondrial DNA–driven activation of the cGAS–STING pathway and interferon secretion

Mitochondrial DNA (mtDNA) functions as a potent mtDAMP that activates cytosolic innate immune sensors. Under conditions of defective mitochondrial vesicle trafficking, altered membrane permeability, or impaired mitophagy, mtDNA can leak into the cytoplasm. The cyclic GMP–AMP synthase (cGAS) recognizes cytosolic mtDNA as aberrant double-stranded DNA and catalyzes the synthesis of 2′3′-cyclic GMP–AMP (2′3′-cGAMP) from ATP and GTP. Acting as a high-affinity ligand, cGAMP binds and activates the endoplasmic-reticulum adaptor STING (stimulator of interferon genes), initiating a downstream signaling cascade (49, 50).

Activated STING recruits and phosphorylates TANK-binding kinase 1 (TBK1), which in turn phosphorylates interferon regulatory factor 3 (IRF3). Phosphorylated IRF3 dimerizes and translocates to the nucleus, where it strongly induces type I interferons (e.g., IFN-α and IFN-β) and a suite of interferon-stimulated genes (ISGs). Concurrently, the STING–TBK1 axis activates the NF-κB pathway through the IKK complex, promoting the expression of multiple proinflammatory cytokines and chemokines. In senescent fibroblasts, sustained activation of the cGAS–STING pathway drives the interferon-related components of the SASP, including CXCL10, ISG15, TNF-α, and IL-6.

Furthermore, mtDNA released into the endolysosomal compartment can be detected by Toll-like receptor 9 (TLR9). Through a MyD88-dependent mechanism, TLR9 signaling enhances NF-κB activation, reinforcing inflammatory amplification within ageing fibroblasts (51). Thus, the cGAS–STING and TLR9 pathways form complementary axes linking mitochondrial distress to chronic interferon and cytokine production.

**Figure 2:**
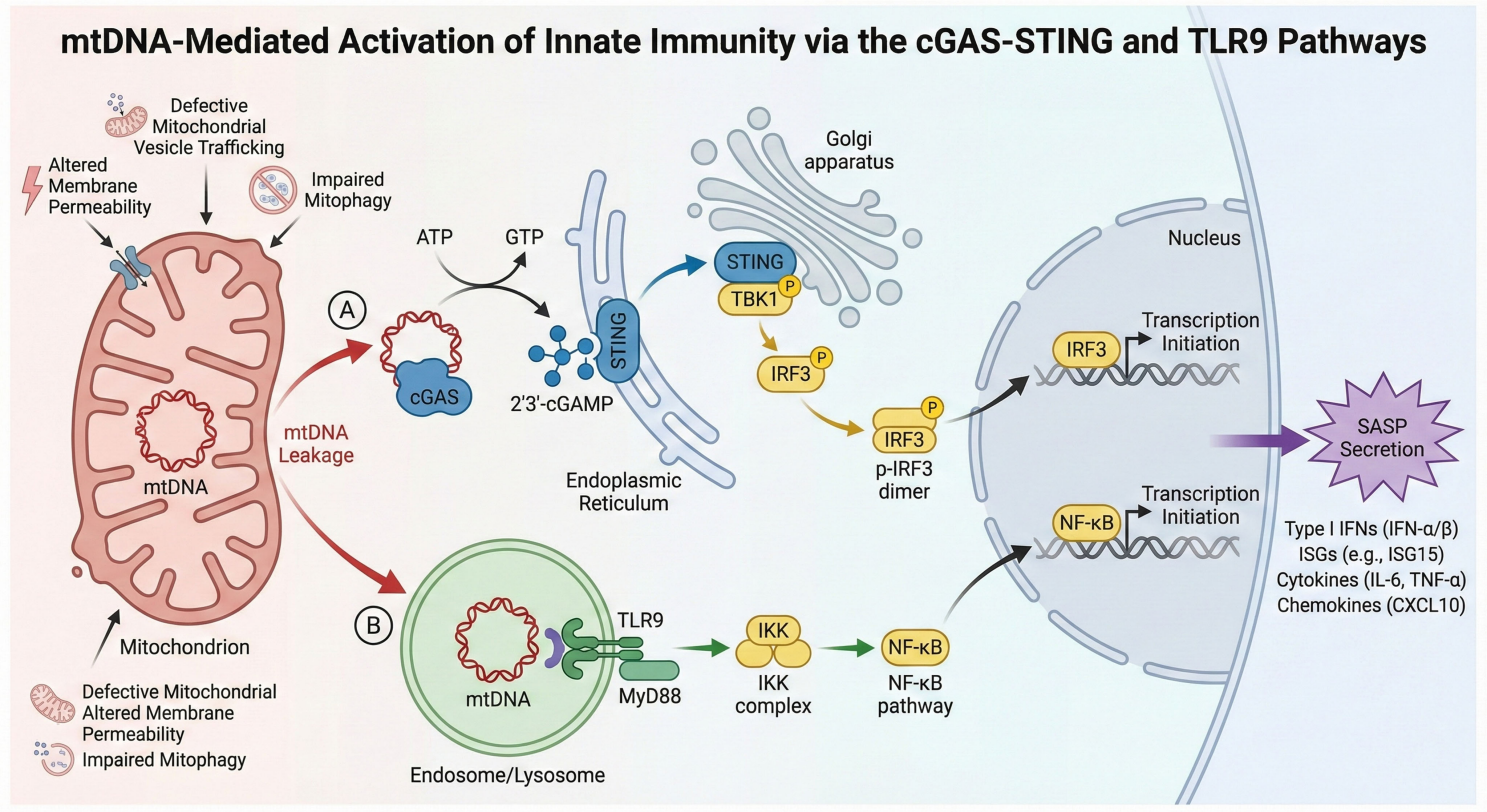
mtDNA-Mediated Activation of Innate Immunity via the cGAS-STING and TLR9 Pathways.

Beyond autocrine signaling, this interferon-rich SASP profoundly shapes fibroblast-immune cell crosstalk within the aging microenvironment. In periodontal tissues, senescent fibroblasts secrete specific chemokines (e.g., CXCL10, IL-8) that act as potent chemoattractants, driving the recruitment and pathological activation of neutrophils. Furthermore, SASP factors skew local macrophage polarization toward a pro-inflammatory M1-like phenotype, while the continuous release of inflammatory cytokines directly stimulates the activation and differentiation of osteoclast precursors. This fibroblast-driven immune mobilization establishes a feed-forward loop of chronic tissue destruction (**Figure 2**).

### 3.3 Oxidized cardiolipin as a cooperative driver of NLRP3 inflammasome activation

Cardiolipin, a mitochondria-specific phospholipid, also contributes to inflammasome activation. Normally confined to the inner mitochondrial membrane, cardiolipin preserves structural integrity and supports oxidative phosphorylation. Mitochondrial injury exposes cardiolipin to the cytosol, where it becomes susceptible to oxidative modification; this can occur through outer membrane rupture or inner membrane inversion. Oxidized cardiolipin can be recognized directly by NLRP3 or indirectly through mitochondrial adaptor proteins, promoting inflammasome assembly and caspase-1–dependent maturation and release of IL-1β and IL-18, thereby exacerbating local inflammatory injury (52–54).

Rather than acting redundantly, excessive mtROS production, aberrant mtDNA release, and cardiolipin oxidation function synergistically and in a highly context-dependent manner. In fibroblasts, mtROS typically serves as the rapid, initiating pulse for NF-κB and inflammasome priming, whereas cytosolic mtDNA establishes a sustained, delayed interferon response via cGAS-STING. Concurrently, oxidized cardiolipin acts as a structural scaffold that further amplifies NLRP3 assembly. While the loss of one signal (e.g., scavenging mtROS) can partially attenuate the acute SASP, the parallel activation of mtDNA and lipid signaling prevents complete compensation, ensuring a robust and irreversible inflammatory response to severe mitochondrial damage. Collectively, these signals constitute a convergent signaling triad that strongly activates the cGAS–STING axis, NLRP3 inflammasome, and NF-κB pathway. These mitochondria-derived danger signals act in concert to initiate and sustain the fibroblast SASP program, establishing a mechanistic link between mitochondrial dysfunction and chronic inflammation during cellular ageing.

It is crucial to distinguish signaling mechanisms validated specifically in fibroblasts from those extrapolated from immune or epithelial models. While certain mitochondrial danger signals act universally, their downstream engagement often exhibits cell-type specificity. The following table summarizes the primary evidence supporting these pathways across different cellular contexts (**Table 1**).

**Table 1:** Comparative Evidence for Mitochondria-Driven Inflammatory Pathways in Fibroblasts and Immune Cells.

Pathway / Mechanism	Evidence in fibroblasts	Evidence in other cell types
mtROS-induced NF-κB Activation	Confirmed (Primary driver of IL-6/IL-8 SASP in stromal models)	Confirmed (Drives acute pro-inflammatory M1 polarization in macrophages)
cGAS-STING Activation via mtDNA	Confirmed (Drives late-stage IFN response in senescent fibroblasts)	Confirmed (Primary DNA-sensing mechanism in myeloid cells)
NLRP3 Inflammasome Activation	Context-dependent (Requires specific epigenetic priming in fibroblasts)	Highly Active (Canonical pyroptosis driver in immune cells)

## 4 Transcriptional logic integrating metabolic and inflammatory signaling

### 4.1 Core transcriptional drivers: cooperative interactions between NF-κB and C/EBPβ

Signals of metabolic dysregulation and mitochondrial damage–associated molecular patterns (mtDAMPs) must ultimately converge in the nucleus, where they are integrated to orchestrate the senescence-associated secretory phenotype (SASP). In fibroblasts, this integration requires highly specific transcriptional regulatory circuits and canonical signaling pathways that collectively determine the composition, magnitude, and persistence of SASP expression (55–57).

Nearly all forms of SASP activation rely on the transcription factor NF-κB, which exhibits the highest basal and inducible activity in fibroblasts. SASP cytokines, particularly TNF-α and IL-1, drive autocrine and paracrine loops that engage their cognate receptors to further amplify NF-κB signaling, thus forming a self-reinforcing circuit. As detailed in Section 2, mitochondrial signaling—via the CGAS-STING-TBK1 cascade and mtROS-mediated IKK oxidation—serves as the primary upstream route liberating NF-κB heterodimers (p65/p50) for rapid nuclear translocation. There, NF-κB binds κB motifs within the promoters of SASP genes such as *IL6*, *IL8*, *CXCL1*, *CXCL2*, *ICAM1*, and *MMPs*, sustaining their transcriptional upregulation. Senescent fibroblasts typically exhibit persistent NF-κB activation, which underlies the long-term stability of SASP output (58–61).

The fibroblast-enriched transcription factor CCAAT/enhancer-binding protein β (C/EBPβ) functions as a key cooperative regulator of *IL6* transcription within the SASP context. Its activity is modulated by MAPK signaling, notably p38 MAPK–mediated phosphorylation. Mitochondrial stress markedly enhances both expression and phosphorylation of C/EBPβ. At the chromatin level, phosphorylated C/EBPβ co-occupies the promoters of *IL6* and *IL8* with NF-κB, forming transcriptional activation complexes that integrate inflammatory and metabolic signals through direct protein–protein interactions, thereby amplifying SASP gene transcription and sustaining the inflammatory circuit (62–64).

### 4.2 Molecular basis of the interferon response: STAT1 and STAT3 as core signaling components

Type I interferons (IFN-α/β) act in both autocrine and paracrine fashions to activate interferon-α/β receptors (IFNAR) on fibroblasts, leading to the recruitment of the receptor-associated Janus kinases JAK1 and TYK2. This process, typically triggered downstream of mtDNA leakage and cGAS–STING activation, initiates STAT1/STAT3 phosphorylation and transcriptional signaling, conferring a characteristic interferon signature upon the fibroblast SASP (65–67).

Activated JAKs phosphorylate STAT1 and STAT3, which then form either homodimers or heterodimers with distinct transcriptional outcomes. Phosphorylated STAT1 homodimers induce interferon-stimulated genes (ISGs) such as *CXCL10* and *ISG15*, hallmarks of the fibroblast SASP. STAT1–STAT3 heterodimers and STAT3 homodimers cooperate with NF-κB to establish a positive regulatory feedback loop that sustains inflammatory signaling and supports cell survival and proliferation. This transcriptional crosstalk ensures persistent *IL6* expression and reinforces the chronic inflammatory microenvironment characteristic of senescent fibroblasts (68, 69).

### 4.3 Metabolic integration hub: reciprocal regulation of the AMPK–mTORC1 axis

The mechanistic target of rapamycin complex 1 (mTORC1) serves as a pivotal platform integrating metabolic and growth-related cues. In ageing fibroblasts, aberrant activation of mTORC1 profoundly influences SASP regulation. By directly enhancing anabolic translation, mTORC1 augments ribosomal capacity and mRNA translation efficiency, thereby promoting the rapid synthesis of SASP proteins such as IL-6 and IL-8 (70, 71).

In addition, mTORC1 indirectly intensifies inflammation through autophagy suppression. Overactivation of mTORC1 disrupts autophagic homeostasis, leading to the accumulation of dysfunctional organelles—including mitochondria—that release mtDAMPs and activate GATA4-dependent transcriptional programs, thereby facilitating SASP initiation. mTORC1-driven metabolic reprogramming also shifts fibroblast metabolism toward biosynthetic pathways, altering NAD^+^ balance and acetyl-CoA availability, which in turn modulate epigenetic and inflammatory signaling intensity (72, 73).

As the physiological antagonist of mTORC1, AMPK plays a central role in maintaining metabolic and inflammatory equilibrium. Upon energy stress, AMPK is activated and phosphorylates Raptor and TSC2, thereby inhibiting mTORC1 activity while promoting autophagic clearance of damaged mitochondria and inflammatory substrates. AMPK also exerts direct or indirect regulatory effects on the NF-κB pathway, forming a dual safeguard mechanism that couples energy conservation with inflammation control. Nevertheless, under chronic, irreversible energy imbalance associated with ageing, the protective capacity of AMPK may become attenuated. Prolonged AMPK activation reflects persistent metabolic stress, which, paradoxically, can reinforce senescence progression and sustain SASP expression (74–76).

Through this integrated transcriptional network, fibroblasts precisely interpret metabolic and stress cues emanating from damaged mitochondria, translating upstream energetic fluctuations into downstream SASP regulation. This metabolic–inflammatory coupling ultimately remodels the tissue microenvironment and perpetuates chronic inflammation.

The manifestation of this transcriptional logic exhibits profound tissue specificity. Compared to dermal fibroblasts, which exhibit a profibrotic SASP rich in TGF-β and collagens, periodontal ligament fibroblasts display a distinct developmental susceptibility to inflammatory degradation. Originating from the cranial neural crest, PDLFs possess intrinsic osteogenic plasticity. Under metabolic stress, this epigenetic plasticity is aberrantly hijacked, resulting in a SASP profile disproportionately enriched in osteoclast-activating factors (such as RANKL) and matrix-degrading enzymes (MMP-8, MMP-13), with a marked absence of compensatory fibrotic repair. This unique metabolic-epigenetic predisposition transforms generic mitochondrial dysfunction into the highly destructive, tissue-specific aging phenotype observed in periodontitis.

## 5 Tissue-specific consequences: the case of periodontal fibroblasts

### 5.1 The periodontal microenvironment induces a distinct SASP signature in ligament fibroblasts

The biological function of the senescence-associated secretory phenotype (SASP) is profoundly shaped by tissue origin and local microenvironmental context. Differences in cellular lineage, functional demand, senescence stimuli, and immune milieu confer high tissue specificity to SASP composition, signaling regulation, and pathological outcomes. Periodontal ligament fibroblasts (PDLFs) in chronic periodontitis represent a paradigmatic model for studying such tissue-specific manifestations of SASP.

Unlike fibroblasts in other organs, periodontal fibroblasts exist in constant contact with a complex oral microbial biofilm. Under homeostatic conditions, they maintain a balanced immune–metabolic state; however, when Gram-negative anaerobes dominate the biofilm, chronic inflammation ensues. Continuous microbial stimulation imposes persistent low-level stress on PDLFs, accelerating senescence (77, 78). Compared with dermal fibroblasts, senescent PDLFs exhibit a highly proinflammatory and tissue-destructive SASP profile, characterized by markedly elevated secretion of IL-1β, IL-6, IL-8, and TNF-α. These cytokines recruit neutrophils and other immune cells, promote osteoclast differentiation and activation, and thus drive alveolar bone resorption. Concurrently, matrix-degrading enzymes—including MMP-1, MMP-2, MMP-3, MMP-8, and MMP-9—are strongly upregulated, causing collagen fiber degradation and extracellular matrix breakdown, which culminate in detachment of the periodontal apparatus. Enhanced chemokine release (CXCL1, CXCL10, MCP-1) further promotes neutrophil and macrophage recruitment, establishing a self-perpetuating inflammatory feedback loop and a cycle of progressive tissue destruction (79–81).

### 5.2 Mitochondrial energetic imbalance as the central mediator of microbial stimulation and SASP induction

Chronic microbial challenge drives SASP activation in PDLFs through mitochondrial dysfunction. Sustained exposure to bacterial lipopolysaccharide (LPS) and proinflammatory mediators induces profound metabolic reprogramming, impairing oxidative phosphorylation (OXPHOS) efficiency while promoting compensatory glycolysis (82). This energetic imbalance represents not a passive degenerative consequence but an active signaling transition. Dysfunctional mitochondria overproduce mtROS, which act as key intermediates linking metabolic stress to inflammatory transcriptional activation. mtROS directly engage NF-κB and NLRP3 inflammasome pathways, initiating transcription and secretion of canonical SASP markers such as IL-1β, IL-6, and MMPs (83).

Notably, mitophagy is markedly impaired in PDLFs as periodontitis progresses, leading to the accumulation of damaged mitochondria that function as inflammatory amplifiers. This accumulation intensifies mtROS generation and mtDNA leakage, fueling a self-reinforcing loop in which mitochondrial dysregulation drives SASP secretion, which in turn escalates inflammation. Such a vicious cycle acts as a principal molecular driver of progressive periodontal tissue destruction (84).

### 5.3 Mechanisms by which SASP exacerbates periodontal tissue damage

The distinct SASP profile of PDLFs contributes to periodontal tissue degradation through multiple synergistic mechanisms. SASP components act in both autocrine and paracrine fashions, stimulating senescent and nonsenescent PDLFs, epithelial cells, and immune cells within the microenvironment. This promotes sustained local inflammation, confers apoptosis resistance, and allows long-term survival of senescent cells that continuously secrete deleterious factors.

**Figure 3:**
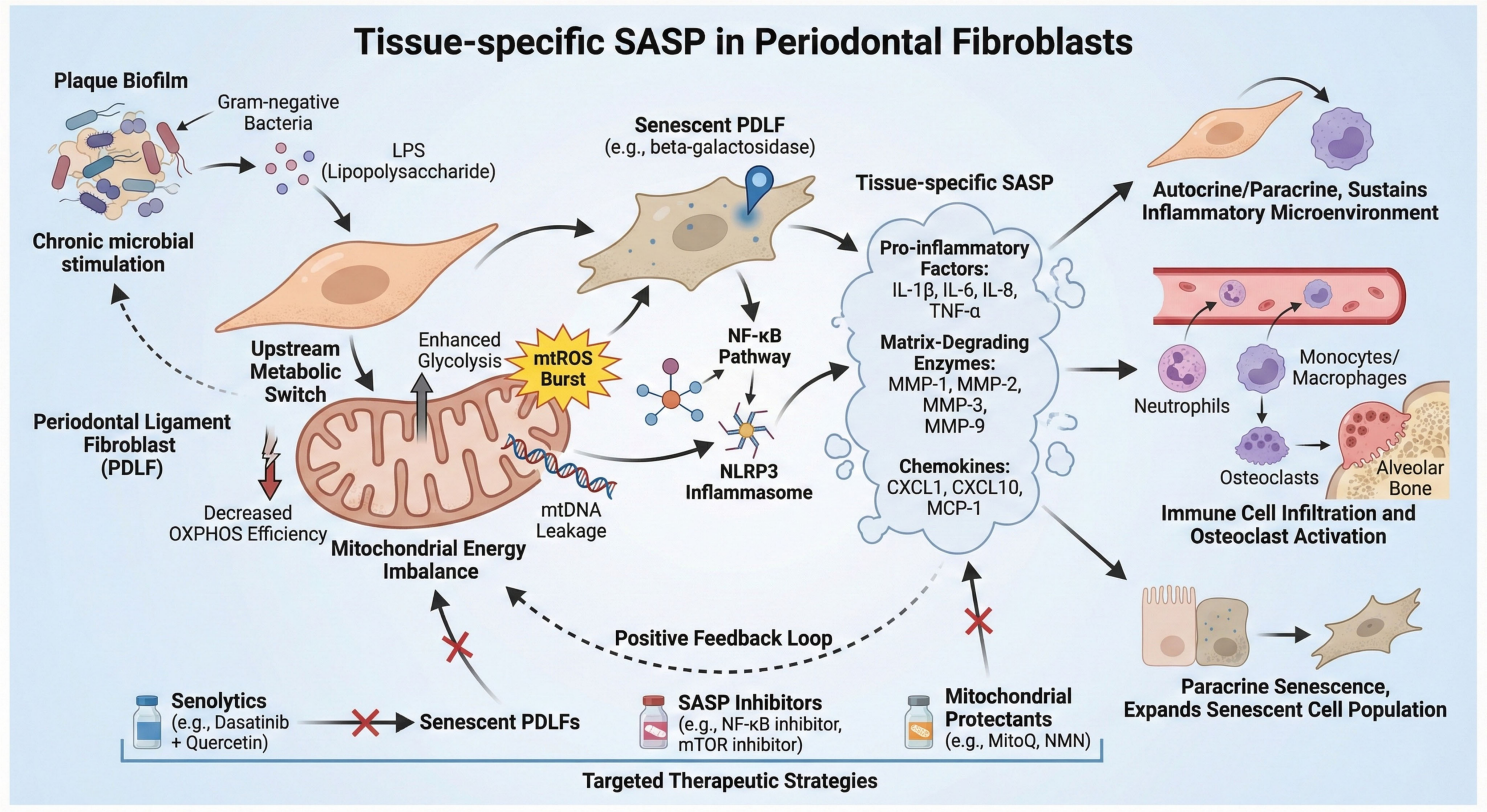
Tissue-specific SASP in Periodontal Fibroblasts.

SASP-derived chemokines attract neutrophils and monocyte/macrophage populations into periodontal pockets, where excessive immune activation leads to protease and reactive oxygen species release, directly exacerbating tissue damage. Cytokines such as RANKL induce osteoclast differentiation from macrophages, facilitating alveolar bone resorption. Moreover, SASP factors propagate senescence spreading, whereby neighboring epithelial, endothelial, and immune cells adopt senescent-like phenotypes, reducing regenerative capacity and further aggravating disease chronicity. This propagation of cellular ageing amplifies the senescent cell pool and entrenches the inflammatory microenvironment (85)(**Figure 3**).

### 5.4 Therapeutic targeting of SASP in periodontal disease: current strategies and future perspectives

Given the central role of SASP in periodontitis pathology, targeting senescent PDLFs and their pathological secretome represents a promising therapeutic frontier. Owing to the anatomic accessibility of periodontal tissues, localized and lesion-specific approaches are particularly advantageous. Senolytic agents such as dasatinib and quercetin selectively induce apoptosis of senescent PDLFs (86, 87). When incorporated into controlled-release platforms—such as periodontal pocket gels or nanocarrier systems—these drugs efficiently eliminate SASP-producing cells at the lesion site, halting the inflammatory cascade at its source.

Senomorphic strategies can further modulate SASP output: NF-κB inhibitors suppress major proinflammatory signaling; JAK inhibitors dampen type I interferon responses; NLRP3 inhibitors reduce IL-1β maturation and release; and mTOR inhibitors such as rapamycin attenuate translation of SASP factors. Upstream mitochondrial interventions also hold promise: mitochondria-targeted antioxidants (e.g., MitoQ) limit mtROS accumulation, whereas NAD^+^ precursors (e.g., NMN) restore sirtuin activity and enhance OXPHOS efficiency, thereby addressing the metabolic origin of fibroblast senescence and mitigating SASP propagation.

Collectively, targeting cellular senescence represents an emerging paradigm in periodontal therapy. SASP reflects the terminal adaptive response of periodontal fibroblasts to chronic microbial stimulation and energetic stress, and its pathological components not only perpetuate inflammation but also impair regeneration. Combining senolytic or senomorphic interventions with mitochondrial metabolic stabilization may therefore effectively disrupt the cascade in which energy failure triggers SASP activation, which then drives chronic inflammation and ultimately leads to tissue destruction. This integrated strategy opens new avenues for periodontal tissue regeneration and functional restoration.

This mechanistic framework dictates a temporally stratified approach to therapeutic intervention. If energy imbalance is the fundamental upstream initiator, early-stage metabolic reprogramming—such as restoring α-KG levels or enhancing NAD+ pools—can effectively preempt SASP activation by re-establishing mitochondrial-nuclear epigenetic communication. However, once mitochondria undergo irreversible structural collapse and massive mtDNA/mtROS leakage occurs, the inflammatory signaling loop becomes self-sustaining and refractory to metabolic correction. At this terminal stage, senolytics become the superior and necessary strategy, functioning to selectively eliminate the source of the inflammatory broadcast rather than attempting to rescue irreparably damaged bioenergetic networks (**Table 2**).

**Table 2:** Therapeutic Strategies Targeting Mitochondrial Dysfunction and SASP in Cellular Senescence.

Intervention category	Representative agents	Primary mechanism of action	Relevance to mitochondrial logic
Senolytics	Dasatinib + Quercetin, ABT-263	Induces apoptosis in senescent cells	Eliminates cells with irreversible bioenergetic collapse
Senomorphics	Rapamycin, Metformin	Suppresses SASP secretion pathways	Modulates the AMPK/mTOR axis to dampen inflammatory output
Mitochondrial Antioxidants	MitoQ, SkQ1	Scavenges mitochondrial superoxide	Prevents mtROS-mediated NF-κB and NLRP3 activation
Metabolic Modulators	α-KG, NAD+ Precursors	Replenishes core metabolic cofactors	Restores epigenetic regulation and mitochondrial metabolic flux
MAM-Targeted Agents	2-APB, Metformin	Modulates ER-mitochondria Ca2+ flux	Prevents pathological tethering and downstream ferroptosis

## 6 Conclusions and perspectives

This review highlights the pivotal role of fibroblasts in organismal ageing, emphasizing how metabolic stress, mitochondrial dysfunction, and innate immune activation jointly drive and sustain the senescence-associated secretory phenotype (SASP). Mitochondria, as central hubs of cellular metabolism and signaling, undergo functional deterioration characterized by reduced ATP synthesis, excessive reactive oxygen species generation, and mtDNA leakage. These events extend beyond secondary consequences of ageing and actively initiate the cascade of inflammatory senescence. Through the integration of fibroblast-specific transcriptional regulators (such as C/EBPβ and STATs) and key metabolic checkpoints (including mTOR and AMPK), mitochondria-derived signals activate canonical innate immune pathways—namely the cGAS–STING axis, the NLRP3 inflammasome, and NF-κB signaling—to generate a complex SASP spectrum composed of cytokines, chemokines, and matrix-degrading enzymes. Collectively, these processes disrupt tissue homeostasis and reinforce chronic inflammation.

From a molecular logic perspective, modulating mitochondrial activity or intercepting its downstream inflammatory pathways offers promising strategies for combating age-related disorders. These interventions encompass several complementary strategies. Senolytic therapies, such as the combination of dasatinib and quercetin, can selectively eliminate senescent cell populations that serve as major SASP sources. Senomorphic approaches employ mTOR, NF-κB, or JAK inhibitors to attenuate SASP production and mitigate its deleterious paracrine effects. In parallel, mitochondria-targeted metabolic interventions, including antioxidants like MitoQ to scavenge mtROS and NAD^+^ precursors like NMN to restore sirtuin activity and OXPHOS efficiency, may prevent the onset of senescence and disrupt the self-perpetuating cycle in which energy imbalance drives SASP activation and chronic inflammation at its origin.

Despite encouraging prospects, significant challenges remain in translating these molecular insights into clinical applications. A deeper understanding of tissue-specific mechanisms—for instance, how localized microbial ecosystems such as the periodontal biofilm modulate SASP composition and kinetics—is essential. Determining the optimal timing and combinatorial strategy for intervention remains a conceptual and technical challenge. Future studies must delineate the fine balance between innate immune defense and chronic inflammation, and explore whether cross-pathway, multicellular coordination is required for effective modulation of senescence-associated inflammation. On the translational front, developing localized drug-delivery platforms, such as controlled-release systems for periodontal lesions, will be critical to achieving high therapeutic efficiency with minimal systemic side effects. Ultimately, a paradigm shift from symptomatic management toward causal intervention at the root of senescence-driven pathology may redefine prevention and treatment strategies for age-related diseases such as periodontitis.
